# Combined presentation of severe pulmonary COVID-19 and multisystem inflammatory syndrome in children (MIS-C): two reported Egyptian pediatric cases

**DOI:** 10.1186/s43054-021-00076-w

**Published:** 2021-12-01

**Authors:** Tarek Hamed, Dina T. Sarhan

**Affiliations:** Pediatric Department, Faculty of Medicine, Zagazig University, Zagazig, Egypt

**Keywords:** Pulmonary, COVID-19, MIS-C, Pediatric, SARS-CoV-2, Egypt

## Abstract

**Background:**

Initial reports from the severe acute respiratory syndrome coronavirus 2 (SARS-CoV-2) pandemic described children as being less susceptible to coronavirus disease (COVID-19) than adults. Later on, a severe and novel pediatric disorder termed multisystem inflammatory syndrome in children (MIS-C) emerged. Pediatric patients with SARS-CoV-2 are at risk for critical illness with severe pulmonary COVID-19 and MIS-C. Both are described as two distinct conditions, and the differentiation between them was the scope of many studies. In this report from Egypt, we will describe two unique pediatric cases presented by combined manifestations of severe pulmonary COVID-19 and MIS-C.

**Case presentation:**

Two patients presented with severe pulmonary COVID-19 evident by pulmonary symptoms, signs, and advanced CO-RADS stage in lung CT were simultaneously fulfilling the clinical criteria of MIS-C including fever, multi-system affection, increased inflammatory markers in addition to the proved COVID-19 by positive serologic tests for SARS-CoV-2 but PCR was negative. Both patients responded well to immune-modulation therapy by IVIG and steroids and discharged well under closed follow-up.

**Conclusions:**

Although it is debatable to present simultaneously, MIS-C should be considered in patients presenting with typical clinical findings and concerns for pulmonary COVID-19 once the criteria for MIS-C diagnosis is fulfilled. Starting treatment without delay can favor better prognosis.

## Background

When the COVID-19 epidemic began in China in late December 2019, pediatric initial reports described an asymptomatic or milder illness in children. In March 2020, the World Health Organization (WHO) declared COVID-19 as pandemic. However, since April 2020, a growing number of reports from different countries have described a severe inflammatory syndrome affecting small number of children [1, 2]. This condition shared features with pediatric other inflammatory conditions in which large amounts of cytokines cause the dysfunction of several organs such as Kawasaki disease (KD) and toxic shock syndrome (TSS) disease [3]. This syndrome has been named multisystem inflammatory syndrome in children (MIS-C). The center for disease control and prevention (CDC) and WHO issued their public health advisory and case definition of MIS-C in May 2020 [4, 5].

MIS-C is defined by clinically severe illness requiring hospitalization with fever, elevated inflammatory marker, and multisystem organ dysfunction in the setting of recent proven or probable COVID-19 infection, and in the absence of an alternative likely explanation [4]. Some of these children may share features of Kawasaki disease, toxic shock syndrome, or cytokine storm syndrome [2]. They can deteriorate rapidly and may need intensive care support as well. The PCR test is more often negative although most of the children have antibodies to SARS-CoV-2. Although the pathogenesis is not clearly known, immune-mediated injury has been implicated [4].

MIS-C appears to be rare but severe, potentially fatal if unrecognized and untreated, it seems to be the most frequent presentation among critically ill children with SARS-CoV-2 infection but may also develop weeks after a mild or asymptomatic SARS-CoV-2 infection. MIS-C patients are older and usually healthy before. They show a higher prevalence of gastrointestinal symptoms and shock and are more likely to receive vasoactive drugs and immunomodulators and less likely to need mechanical ventilation than non-MIS-C patients [6].

It is unknown if the processes that mediate MIS-C are similar to the processes that lead to life-threatening respiratory failure and shock with COVID-19 [7]. Acute COVID-19 and MIS-C are described as two distinct conditions and it is unusual to present simultaneously.

In this report from Egypt, we describe two unique pediatric patients presented by combined manifestations of severe pulmonary COVID-19 and MIS-C. Written informed consents were obtained from their parents before data collection.

## First case presentation

A 10-years-old previously healthy female patient presented to the emergency room (ER) in February 2021 with hypotension and shock. She reported previous 5 days of acute high grade fever and dry cough followed by gastrointestinal symptoms in the form of frequent vomiting and abdominal pain. She had no recent travel but her grandfather was ventilated because of severe COVID-19 pneumonia. She was not on any chronic medications and had no known allergies. She was treated by paracetamol and cough sedatives over the prior 5 days for symptomatic relief.

On presentation, she appeared ill and dyspneic. She was oriented but agitated, febrile (temperature was 39 °C), hypotensive (blood pressure was 70/40 mmHg), and hypoxemic (oxygen saturation on room air was 85%). She had tachypnea and tachycardia. By auscultating the chest, there was diminished air entry and bilateral crepitation. Abdominal examination revealed nothing of note apart from tenderness all through.

Laboratory work-up initially was notable for leukocytosis and lymphopenia with high neutrophil/lymphocyte ratio (NLR). Different inflammatory markers were increased: CRP, ferritin, and LDH. Procalcitonin was also high in spite that both blood and sputum cultures showed no growth. Also, there was documented hyper-coagulable state as D-dimer was more than eight folds the normal value, but Doppler ultrasound studies were negative for thrombi, cardiac affection was evident by laboratory results such as serum troponin and CPK were elevated. All the laboratory results are illustrated in Table 1.

**Table 1:** Laboratory results of the first case on admission, follow-up, and at discharge

Investigations			First case				
			On admission	Days			At discharge
				3	6	8	
**Labs**	**CBC**	**TLC** (×10^9^/L)	**26.300**	**20.2**	12.6	9.2	7.2
		**Lymphocytes** (×10^9^/L)	**0.8**	**1.1**	**1.5**	2	2.1
		**Neutrophils** (×10^9^/L)	**24.100**	**18.1**	10.3	6.5	4.5
	**Inflammatory markers**	**- CRP** (mg/dl)	**168**	**203**	35	20	19
		**- Procalcitonin** (ng/ml)	**3.84**	/	1.1	/	0.5
		**- LDH** (U/L)	**367**	**370**	235	167	110
		**- Ferritin** (μg/dl)	**4738**	**1340**	**290**	**250**	224
		**- IL 6 (**pg/mL)	**10.5**	/	3.26	/	2.7
		**- D-dimer** (mcg/mL)	**4.4**	**3.2**	**1.38**	**1.2**	0.8
	**Cardiac enzymes**	**-Troponin** (ng/mL)	**0.65**	0.26	0. 07	0.037	0.025
		**-CKMB** (U/L)	/	**8.6**	3.5	1.6	0.83
		**-CPK** (U/L)	**218**	**110**	70	15	16
	**Organ function tests**	**-Creatinine** (mg/dL)	0.6	0.5	0.5	0.5	0.5
		**- ALT** (IU/L)	19	23	22	23	24
		**- Albumen** (g/dL)	**2.7**	**2.6**	**2.9**	**2.9**	**2.9**
	**SARS-CoV-2**	**Ig G**	**+**	/	/	/	/
		**Ig M**	**+**	/	/	/	/
		**PCR**	−	/	/	/	/

NB: Bold means significant values in diagnosis (see text). Daily laboratory evaluation was performed. Only data of significant changes was mentioned

**Fig. 1:**
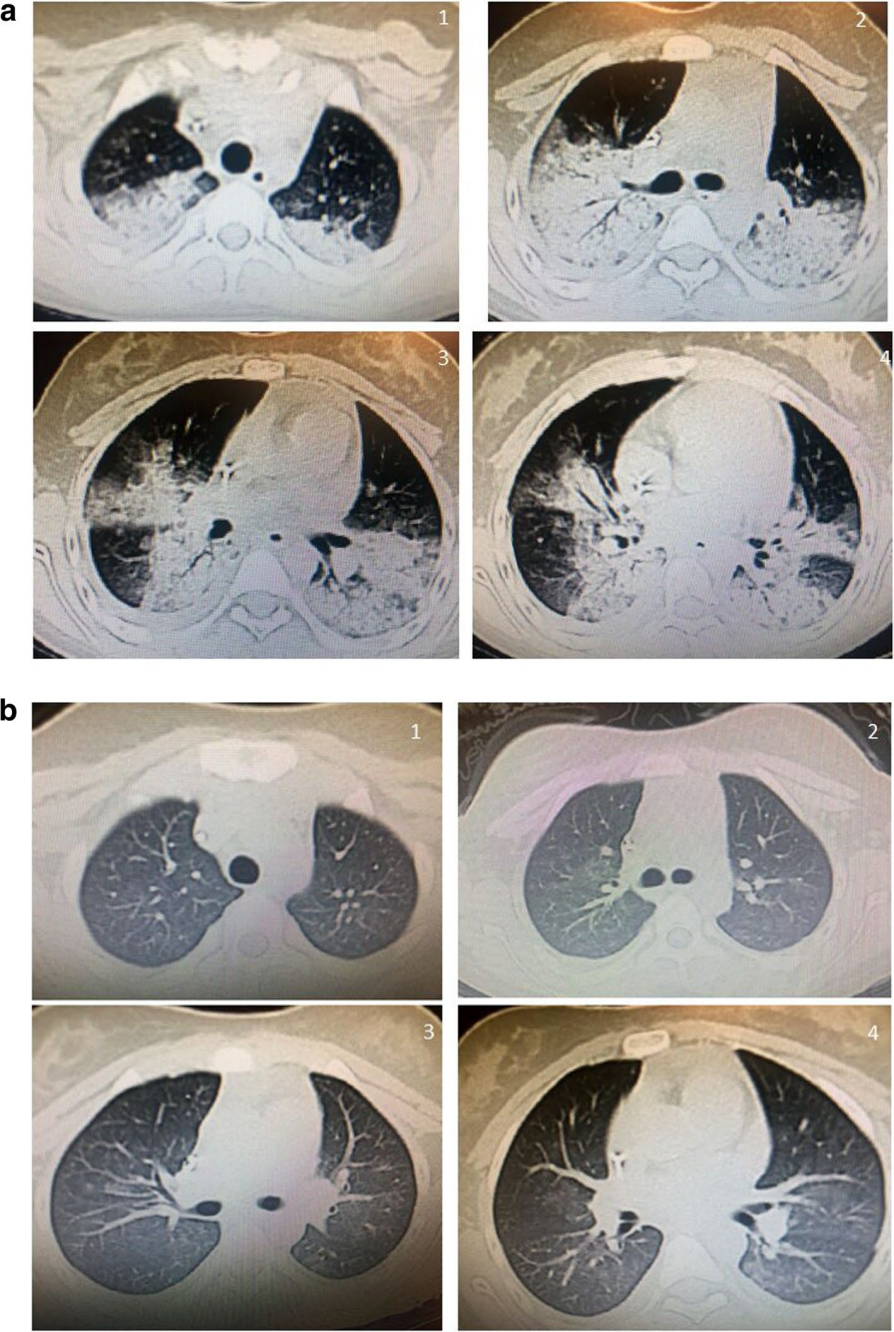
**a** Case 1 initial serial CT cuts showing the large bilateral pulmonary predominantly posterior diffuse areas of ground-glass opacities and consolidations with related accentuated vascularity and septal thickening (CO-RADS 5). **b** Follow-up case 1 serial CT cuts showing significant regressive course with near total resolution but still seen bilateral diffuse mosaic attenuation of ground glass pattern

SARS-CoV-2 PCR from nasopharyngeal swab was negative but SARS-CoV-2 IgG and IgM from serum were positive. Chest CT without contrast showed picture of typical COVID-19 affection in the form of large bilateral pulmonary predominantly posterior sub-pleural diffuse areas of ground-glass opacities and consolidations with related accentuated vascularity and septal thickening (CO-RADS 5) (Fig. 1).

Workup for the shock revealed evidence of worsening cardiac dysfunction. An echocardiogram revealed markedly decreased myocardial contractility and a point of care was the dilated inferior vena cava (IVC).

The patient was admitted to the intensive care unit (ICU) for hypotension and shock, with diagnosis of pulmonary COVID-19 and concern for possible MIS-C according to the case definition of CDC [4] due to gastrointestinal and cardiac systems involvement along with elevated inflammatory markers not linked to other obvious inflammation or infection than COVID-19 (as all cultures were negative).

The patient maintained normal oxygenation on high flow nasal cannula with prepress valve to give oxygen at a rate of 20 L/min. She also received inotropes in the form of dopamine till blood pressure was raised enough to establish central line then she was shifted to noradrenalin and milrinone. In spite of being shocked, we have to add furosemide cautiously to decrease the preload—evidenced by engorged IVC—on the poorly contracting heart, in addition to fluid supplementation guided by monitored cardiac function by ECHO, central venous pressure, and urine output. Antibiotics were given to control infection until results of cultures appeared.

As we consider the diagnosis of MIS-C, treatment with intravenous immunoglobulin (IVIG) was started at a dose of 2 g/kg, but the dose was divided and given on 2 days as the patient’s impaired cardiac function would not tolerate the volume overload of the single infusion. Simultaneously, the patient started pulse methyleprednisolone doses (30 mg/k/day) on three successive days followed by 2 mg/kg/day on two divided doses.

Fever declined 36 h after starting immunomodulating therapy, but she developed severe headache which is documented complication of IVIG therapy. We took in our consideration that this patient had high D-dimer which indicates tendency for thrombosis, so brain MRA was urgently done to exclude cerebral thrombosis and it was free and the headache improved by analgesics within 2 days. Moreover, due to the severe dyspnea, tachypnea, and tachycardia at day of presentation, pulmonary CT angiography was performed to exclude pulmonary embolism. As there was no clinical or radiologic evidence of thrombosis present; a prophylactic dose of subcutaneous low molecular weight heparin (0.5 mg/kg/12 h) was started.

Her leukocytosis and lymphopenia began to downtrend on hospital day 3, and the elevated inflammatory markers also started to decline (Table 1 shows the initial, follow-up, and at discharge laboratory data). Clinical improvement included gradual lowering of oxygen flow became stable on room oxygen on hospital day 7 of admission with significant regression of the initial pulmonary CT findings, and gradual withdrawal of inotropes to be stopped on day 6 of admission. She was discharged from hospital on day 10 on oral steroids and anticoagulants with COVID-19 clinic follow-up plan.

## Second case presentation

One-year-old previously healthy female patient presented with fever, generalized convulsions, and disturbed conscious level. She reported previous 3 days of acute high grade fever and dry cough followed by gastro-intestinal symptoms in the form of vomiting and diarrhea and then the neurologic symptoms developed in the form of convulsion at the day of admission.

**Fig. 2:**
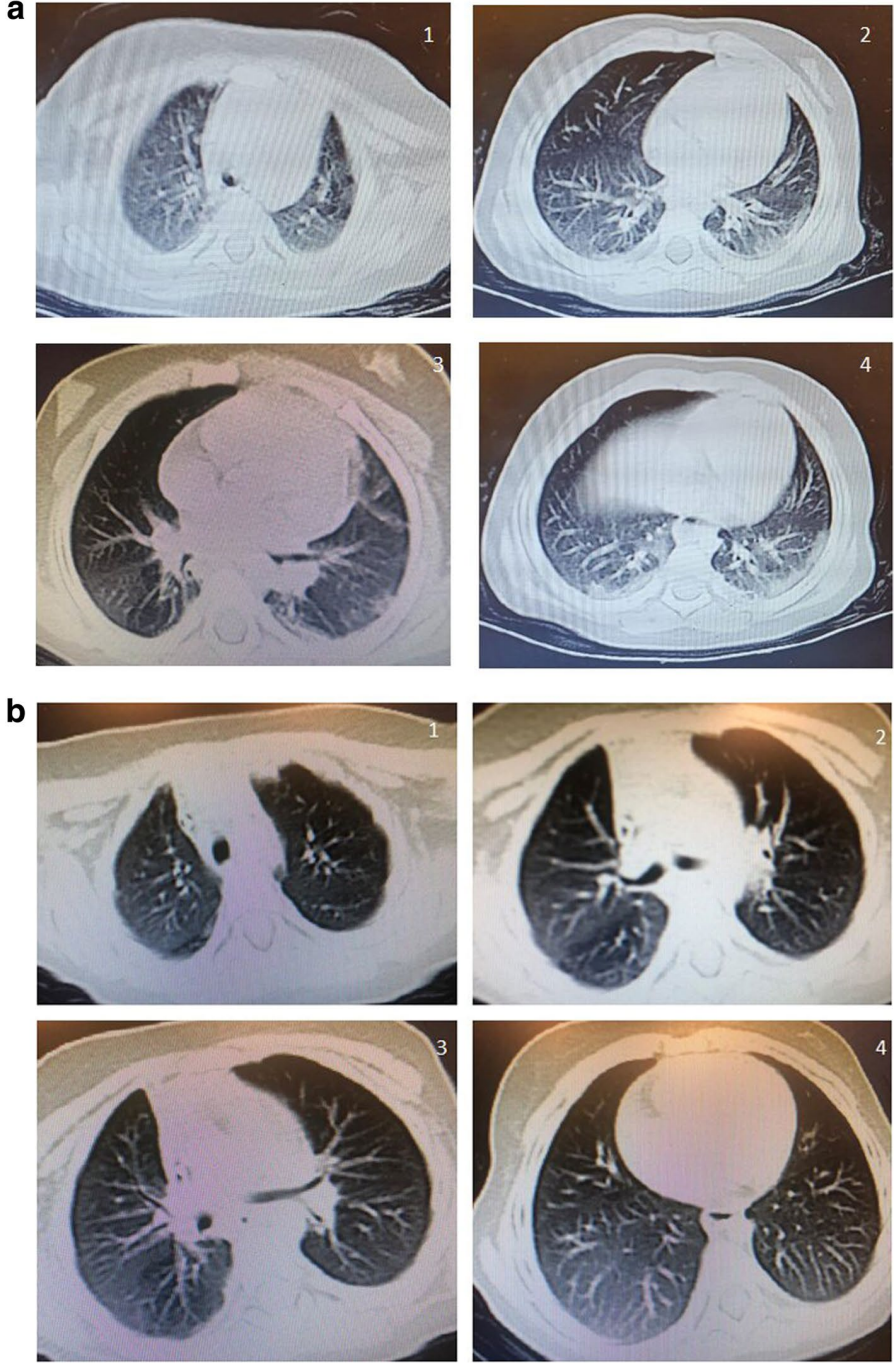
**a** Case 2 initial serial CT cuts showing bilateral multiple pulmonary asymmetric variably defined multilobar areas of ground-glass opacities predominantly peripheral with sub-pleural posterior consolidations with related accentuated vascularity and air space pattern involving all both lung lobes (CO-RADS 4). **b** Follow-up case 1 serial CT cuts showing significant regressive course with marked resolution, but still noted ground glass veiling and bronchial thickening denoting post inflammatory small airway disease sequels

At presentation, the patient was feverish (T° was 39 °C), dyspneic, mildly dehydrated, hypotensive, and in postictal confusion. By examination, she had tachypnea and the chest auscultation revealed diminished air entry and bilateral crepitation. Apart from tachycardia, there was no abnormality on cardiac examination. The patient was admitted to PICU and managed at first as meningitis, there was initial leukocytosis with shift to the left, normocytic normochromic anemia, and high CRP but the CSF examination and brain MRI were normal in absence of meningeal irritation signs. By laboratory follow-up, on the third day of admission, TLC started to decrease and showed lymphopenia, with continuing elevation of inflammatory markers: CRP and LDH in addition to CPK and CKMP levels. At that time, the patient was still feverish, dyspnea decreased on oxygen by nasal catheter which gave oxygen at a rate of 6 L/min, still tachypneic but no more convulsions. Her initial pulmonary CT revealed picture suggestive of COVID-19 infection; there were bilateral multiple pulmonary multi-lobar areas of ground-glass opacities predominantly peripheral with sub-pleural posterior consolidations with related accentuated vascularity and air space pattern involving all both lung lobes (CO-RADS 4) (Fig. 2) while cardiac evaluation by echocardiography showed acute cardiac dysfunction in the form of impaired myocardial function. RT-PCR for SARS-CoV-2 was negative but specific IgG antibodies were detected high by serologic test. In spite, she gave no history of contact with any sick person within the last weeks.

This patient fulfilled the criteria of MIS-C according to the CDC case definition including fever in the involved age with severe illness indicated ICU admission affecting four systems with rising inflammatory markers and evidence of COVID-19 diagnosis, so IVIG was given divided on 2 days because of impaired cardiac function in addition to low dose methyleprednisolone (2 mg/kg/day) and the fever subsided for 24 h after the first dose of IVIG. Recurring of fever after one afebrile day along with rising levels of inflammatory markers (on day 5 of admission as shown in Table 2) indicated giving pulse doses of methyleprednisolone (30 mg/kg/day) for three successive days with permanent regression of fever and clinical improvement of symptoms with decreasing inflammatory markers within 1 week duration. She was discharged from hospital on day 10 on oral steroids with COVID-19 clinic follow-up (Table 2 shows the initial, follow-up, and on discharge laboratory results). She did not need anticoagulant prophylaxis as her D-dimer did not exceed double the normal value throughout her illness. Her follow-up CT of the chest showed significant regressive course with marked resolution, but still noted ground glass veiling and bronchial thickening denoting post inflammatory small airway disease sequels.

**Table 2:** Laboratory results of the second case: on admission, follow-up, and at discharge

Investigations			Second case				
			On admission	Day			At discharge
				3	5	7	
**Labs**	**CBC**	**TLC** (×10^9^/L)	**30.4**	15.7	8.8	3.7	8.5
		**Lymphocytes** (×10^9^/L)	8.6	**1.9**	**0.8**	**1.4**	2.2
		**Neutrophils** (×10^9^/L)	**17.9**	13.4	7.7	3.3	5.7
	**Inflammatory markers**	**- CRP** (mg/dl)	**30**	**37**	26	13	1.3
		**- Procalcitonin** (ng/ml)	**7.15**	**5.09**	1.9	1.7	0.33
		**- LDH** (U/L)	299	**388**	**444**	**424**	338
		**- Ferritin** (μg/dl)	30	**55**	**60**	44	48
		**- IL 6 (**pg/mL)	1.9	2.1	/	/	/
		**- D-dimer** (mcg/mL)	0.8	0.7	0.9	0.9	0.9
	**Cardiac enzymes**	**-Troponin** (ng/mL)	0.16	0.022	0.08		0.043
		**-CKMB** (U/L)	3.8	/	**7.1**	**4.2**	2
		**-CPK** (U/L)	115	**983**	**458**	**224**	81
	**Organ function tests**	**-Creatinine** (mg/dL)	0.4	0.4	0.5	0.3	0.4
		**- ALT** (IU/L)	24	24	25	23	21
		**- Albumen** (g/dL)	**2.6**	**2.3**	2.8	3	3.1
	**SARS-CoV-2**	**Ig G**	**+**	/	/	/	/
		**Ig M**	−	/	/	/	/
		**PCR**	−	/	/	/	/

NB: Bold means significant values in diagnosis (see text). Daily laboratory evaluation was performed. Only data of significant changes was mentioned

## Discussion

Although SARS-CoV-2 has not been definitively proven as the cause of MIS-C, the fact that MIS-C appeared during outbreaks of COVID-19 in Europe and the USA is highly suggestive. If the condition becomes less common as the pandemic terminates, it will further support the relationship [8].

The CDC case definition of MIS-C is extremely broad and would be met in many infectious and inflammatory conditions of childhood including acute COVID-19, KD, viral infections, systemic onset juvenile idiopathic arthritis, toxic shock syndrome, and HLH. The course of MIS-C is generally favorable if timely and proper management is provided. Such hyperinflammatory syndrome is different in presentation and management than the classic COVID-19 disease with severe respiratory involvement [8].

Fever is the cardinal symptom in patients with MIS-C, with 100% of patients developing a fever, whereas a third of the classic SARS-CoV-2 patients without MIS-C do not present a fever. This syndrome is characterized by gastrointestinal symptoms, cutaneous and may be neurologic manifestations with elevation of inflammatory markers, but the most serious are hypotension, shock, and acute cardiac dysfunction. There were a high proportion of MIS-C patients requiring vasoactive drugs even with conserved cardiac function which may indicate an important element of vasoplegia in these patients [6].

It should be remembered that acute COVID-19 can also affect multiple organ systems including increased coagulation tendency [9].

Although elevation of inflammatory markers is detected in patients with COVID-19, patients with MIS-C exhibit lower levels of lymphocytes and LDH, higher levels of CRP and PCT, neutrophils, and a higher lymphocyte/neutrophil ratio in addition to the higher level of cardiac troponin.

In adults, hyper-inflammation is more frequent in the context of COVID-19 bilateral pneumonia, while in children it is more frequent in patients with mild or absent respiratory symptoms presenting gastrointestinal symptoms and shock fulfilling MIS-C criteria, whereas patients with classic SARS-CoV-2 typically show respiratory symptoms [6].

The overlap between the two conditions—COVID-19 and MIS-C—was described as differing manifestations of the new clinical syndrome in a latent class analysis study published by the CDC as initial findings of 570 children in the USA. The study divided patients of MIS-C into three class groups based on the shared characteristics with KD and acute COVID-19; in their described class 2, the clinical and laboratory manifestations of MIS-C overlapped with those in acute COVID-19. These patients had the most respiratory symptoms and highest prevalence of nasopharyngeal RT-PCR positivity for SARS-CoV-2, and likely had acute COVID-19 [10]. This was similar to the presentation of our patients while many other studies reported that MIS-C can develop weeks after COVID-19 infection, suggesting its immune-mediated cause [3, 11, 12].

In our two reported cases, there were combined manifestations of severe pulmonary COVID-19 evidenced by the respiratory symptoms along with the extensive pulmonary affection in the CT (CO-RADS 4 and 5) (described in Figs. 1 and 2), and manifestations of severe MIS-C evidenced by the critical presentation of shock in the first case and picture of infection in the second one in addition to compromising cardiac functions evidenced by echocardiography and elevated cardiac enzymes level. Moreover, all the criteria of MIS-C according to CDC case definitio n[4] were fulfilled; fever, multi-system affection, increased markers of inflammation, evidence of COVID-19 (positive serology for SARSE-CoV-2) with no other obvious microbial cause of inflammation (where all cultures were negative). This overlap might result from the development of MIS-C soon after symptomatic acute COVID-19 illness.

In our two patients, the manifestations of both conditions, COVID-19 and MIS-C came simultaneously, but both had negative RT-PCR from nasopharyngeal swab while tested COVID-19 positive by serology in contradictory to class 2 described by CDC where nasopharyngeal swab PCR reported positive [6]. CO-RADS 5 in patient’s CT was a supporting evidence for diagnosis of COVID-19 in spite of the negative PCR. The majority of published cases of MIS-C have had positive serologic testing for SARS-CoV-2 and less commonly positive RT-PCR testing from nasopharyngeal testing, suggesting that this syndrome may be post-infectious and associated with lower viral burden when compared with acute early infection. Other opinions suggests that MIS-C may be caused by an underlying deregulation of the immune system, with the viral infection triggering a hyperinflammatory response rather than being a direct expression of SARS-CoV-2 infection [1, 13].

So, we have two unique cases different from the classic severe pulmonary COVID-19, classic MIS-C, and even different from the CDC described class of overlap between them.

The outcome of both patients was generally favorable; they were discharged from hospital within 10 days with follow-up at the COVID-19 outpatient clinic.

## Conclusions

Although it is debatable to present simultaneously, MIS-C should be considered in patients presenting with typical clinical findings and concerns for pulmonary COVID-19 once the criteria for MIS-C diagnosis is fulfilled. Starting treatment without delay can favor better prognosis.

## Data Availability

All data generated or analyzed during this study are included in this published article. Any other information is available from authors on reasonable request.

## Abbreviations

CDC: The Center for Disease Control and Prevention
CO-RADS: The COVID-19 Reporting and Data System
COVID-19: Coronavirus disease 2019
CPK: Creatine phosphokinase
CRP: C-reactive protein
CSF: Cerebrospinal fluid
CT: Computed tomography
HLH: Hemophagocytic lymphohistiocytosis
ICU: Intensive care unit
IL: Interleukin
IVC: Inferior vena cava
IVIG: Intravenous immunoglobulin
KD: Kawasaki disease
LDH: Lactate dehydrogenase
MIS-C: Multisystem inflammatory syndrome in children
MRA: Magnetic resonance angiography
MRI: Magnetic resonance imaging
NLR: Neutrophil/lymphocyte ratio
PCT: Procalcitonin
RT-PCR: Reverse transcription polymerase chain reaction
SARS-CoV-2: Severe acute respiratory coronavirus 2
WHO: World Health Organization
